# 554 Implementation of a Checklist in Burn Surgery: Usage Improves Operation Efficiency and Cost

**DOI:** 10.1093/jbcr/irad045.150

**Published:** 2023-05-15

**Authors:** Douglas Bettarelli, Vishal Bandaru, Khaja Siddiqui, Travis Cole, Chip Shaw, Deepak Bharadia, Alan Pang, John Griswold

**Affiliations:** TTUHSC SOM, Lubbock, Texas; TTUHSC SOM, Lubbock, Texas; TTUHSC SOM, Lubbock, Texas; TTUHSC, Lubbock, Texas; Texas Tech University Health Sciences Center, Lubbock, Texas; Texas Tech University Health Sciences Center, Lubbock, Texas; Texas Tech University Health Sciences Center, Lubbock, Texas; Texas Tech University Health Sciences Center, Lubbock, Texas; TTUHSC SOM, Lubbock, Texas; TTUHSC SOM, Lubbock, Texas; TTUHSC SOM, Lubbock, Texas; TTUHSC, Lubbock, Texas; Texas Tech University Health Sciences Center, Lubbock, Texas; Texas Tech University Health Sciences Center, Lubbock, Texas; Texas Tech University Health Sciences Center, Lubbock, Texas; Texas Tech University Health Sciences Center, Lubbock, Texas; TTUHSC SOM, Lubbock, Texas; TTUHSC SOM, Lubbock, Texas; TTUHSC SOM, Lubbock, Texas; TTUHSC, Lubbock, Texas; Texas Tech University Health Sciences Center, Lubbock, Texas; Texas Tech University Health Sciences Center, Lubbock, Texas; Texas Tech University Health Sciences Center, Lubbock, Texas; Texas Tech University Health Sciences Center, Lubbock, Texas; TTUHSC SOM, Lubbock, Texas; TTUHSC SOM, Lubbock, Texas; TTUHSC SOM, Lubbock, Texas; TTUHSC, Lubbock, Texas; Texas Tech University Health Sciences Center, Lubbock, Texas; Texas Tech University Health Sciences Center, Lubbock, Texas; Texas Tech University Health Sciences Center, Lubbock, Texas; Texas Tech University Health Sciences Center, Lubbock, Texas; TTUHSC SOM, Lubbock, Texas; TTUHSC SOM, Lubbock, Texas; TTUHSC SOM, Lubbock, Texas; TTUHSC, Lubbock, Texas; Texas Tech University Health Sciences Center, Lubbock, Texas; Texas Tech University Health Sciences Center, Lubbock, Texas; Texas Tech University Health Sciences Center, Lubbock, Texas; Texas Tech University Health Sciences Center, Lubbock, Texas; TTUHSC SOM, Lubbock, Texas; TTUHSC SOM, Lubbock, Texas; TTUHSC SOM, Lubbock, Texas; TTUHSC, Lubbock, Texas; Texas Tech University Health Sciences Center, Lubbock, Texas; Texas Tech University Health Sciences Center, Lubbock, Texas; Texas Tech University Health Sciences Center, Lubbock, Texas; Texas Tech University Health Sciences Center, Lubbock, Texas; TTUHSC SOM, Lubbock, Texas; TTUHSC SOM, Lubbock, Texas; TTUHSC SOM, Lubbock, Texas; TTUHSC, Lubbock, Texas; Texas Tech University Health Sciences Center, Lubbock, Texas; Texas Tech University Health Sciences Center, Lubbock, Texas; Texas Tech University Health Sciences Center, Lubbock, Texas; Texas Tech University Health Sciences Center, Lubbock, Texas; TTUHSC SOM, Lubbock, Texas; TTUHSC SOM, Lubbock, Texas; TTUHSC SOM, Lubbock, Texas; TTUHSC, Lubbock, Texas; Texas Tech University Health Sciences Center, Lubbock, Texas; Texas Tech University Health Sciences Center, Lubbock, Texas; Texas Tech University Health Sciences Center, Lubbock, Texas; Texas Tech University Health Sciences Center, Lubbock, Texas

## Abstract

**Introduction:**

Checklists have been successfully implemented in operating rooms (OR) around the United States to improve communication, efficiency, and patient well-being. They can be relatively simple additions to facilitate communication and prepare operating rooms before surgeries. Even so, team member buy-in is paramount for physicians to maintain consistent usage when operating. Systematizing actions can be challenging in emergent situations, so effective strategies to ensure timely and beneficial patient outcomes is critical. Proper checklist execution has dramatically improved patient outcomes and operation efficiency, namely those in operating rooms with complex surgeries. We hypothesized that a checklist would reduce the average time of surgeries in a burn unit.

**Methods:**

We analyzed how checklist implementation impacted surgery duration and time spent in an OR. Our curated checklist used TeamSTEPPS strategies to communicate predicted surgery sites, supplies, expectations, and concerns. We collected retrospective data from operations in the burn unit one year before the checklist (August 18, 2020 – August 18, 2021) and one year after the checklist (August 19, 2021 – August 19, 2022). Patients chosen were diagnosed with second or third-degree burns and operated on by one of three burn surgeons in the hospital using the checklist. We categorized operations based on burn size based on total body surface area (TBSA), body square area operated on, and procedures with debridement, grafting, or both to ensure similar patient populations.

**Results:**

Our current data consists of operations before (n=142) and after (n=139) the checklist was implemented, respectfully. The average time of surgery was 106.6 minutes before and 93.7 minutes after the checklist. The average time in the operating room per operation was 166.1 minutes before and 155.1 minutes after the checklist.

**Conclusions:**

We learned that average surgery length decreased by 13.0 minutes (12.1%) and patient OR time decreased by 10.9 minutes (6.6%) with p-values of 0.013 and 0.053, respectively. We plan to expand the range of patients further and look at costs saved in the operating room.

**Applicability of Research to Practice:**

The World Health Organization (WHO) advocates for specialized checklists in operating rooms that encounter complex surgeries with intensive care because of their notable impact on efficiency. Based on WHO’s checklist recommendation, burn units may significantly improve due to the many complications that arise during operations. Complicated issues during debridement and grafting include hypothermia, infection, inflammatory response, and systemic shutdown. Additionally, burn patients may require several nonelective surgeries to have the best outcome, adding another layer of complexity. When used in these clinical events, a checklist can encourage preparedness and communication that may improve operation times and cost of care.

